# Graphene-Oxide and Ionic Liquid Modified Electrodes for Electrochemical Sensing of Breast Cancer 1 Gene

**DOI:** 10.3390/bios12020095

**Published:** 2022-02-04

**Authors:** Deniz Işın, Ece Eksin, Arzum Erdem

**Affiliations:** Analytical Chemistry Department, Faculty of Pharmacy, Ege University, Bornova, Izmir 35100, Turkey; denizisin4@gmail.com (D.I.); eceksin@hotmail.com (E.E.)

**Keywords:** graphene oxide, ionic liquid, electrochemical nucleic acid sensor, breast cancer 1 gene, DNA hybridization

## Abstract

Graphene-oxide and ionic liquid composite-modified pencil graphite electrodes (GO-IL-PGEs) were developed and used as a sensing platform for breast cancer 1 (BRCA1) gene detection. The characterization of GO-IL modified electrodes was executed by scanning electron microscopy (SEM), cyclic voltammetry (CV), and electrochemical impedance spectroscopy (EIS). The nucleic-acid hybridization was monitored by a differential pulse voltammetry (DPV) technique by directly measuring the guanine oxidation signal without using any indicator. The effects of the IL concentration, the probe concentration, and the hybridization time were optimized to the biosensor response. The limit of detection (LOD) was calculated in the concentration range of 2–10 μg/mL for the BRCA1 gene and found to be 1.48 µg/mL. The sensitivity of the sensor was calculated as 1.49 µA mL/µg cm^2^. The developed biosensor can effectively discriminate the complementary target sequence in comparison to a three-base-mismatched sequence or the non-complementary one.

## 1. Introduction

Breast cancer is one of the most serious types of cancer affecting human health worldwide. The region of human chromosome 17q12-21, which contains the breast-cancer susceptibility gene known as BRCA1, is responsible for approximately 80% of breast-cancer families and ovarian cancer and is also associated with other types of cancer, such as pancreatic and colon cancers. BRCA1 encoded a tumor-suppressor protein that controls the cell cycle, repairs damaged DNA, and maintains genomic stability. Mutations in BRCA1 affect the proteins produced, resulting in an increased risk of breast cancer. Besides BRCA1 mutation, the alteration of the gene-expression level can also affect biological processes and contribute to both sporadic and hereditary breast-tumor progression [1].

Breast cancer is one of the most serious types of cancer affecting human health worldwide. The region of human chromosome 17q12-21, which contains the breast-cancer susceptibility gene known as BRCA1, is responsible for approximately 80% of breast-cancer families and ovarian cancer and is also associated with other types of cancer, such as pancreatic and colon cancers. BRCA1 encoded a tumor-suppressor protein that controls the cell cycle, repairs damaged DNA, and maintains genomic stability. Mutations in BRCA1 affect the proteins produced, resulting in an increased risk of breast cancer. Besides BRCA1 mutation, the alteration of the gene-expression level can also affect biological processes and contribute to both sporadic and hereditary breast-tumor progression [1].

Conventional methods such as capillarity electrophoresis [2], sequencing [3,4], and PCR-mediated techniques [5,6] are available for analyzing cancer-related genes. Even though these methods are sensitive, there are some limitations, such as high costs, time consuming, and complex experimental steps with harmful chemical use and the requirement of highly trained staff. Thus, it is urgent to develop a low-cost, fast, and sensitive detection method for the analysis of cancer-related gene sequences. In this context, nucleic acid biosensors provide a simplified method to detect cancer related nucleic-acid sequences [1,7,8,9,10,11,12].

Recently, the fluorescent method has become popular in the diagnosis of cancer or tumor tissues due to its advantages of sensitivity and specificity. For instance, Liu et al. developed a fluorescent method based on hairpin DNA-templated copper nanoclusters (CuNCs) for the detection of the BRCA1 gene [10]. Although the fluorescent sensor for the BRCA1 gene assay showed good assay performance, fluorescent-based assays also have some limitations, such as limited applications, being labor-intensive (i.e., labelling molecules), and validating tools often being required. On the other hand, surface plasmon resonance biosensors can provide an alternative platform for detection of point mutations. For example, Li et al. presented a method that combines primer extension analysis with SPR to discriminate single-base mutations related to the BRCA1 gene [11]. One potential limitation of the SPR method is that the ligand may not maintain its native configuration upon immobilization on the sensor chip surface, or its orientation may sterically hinder analyte binding. Non-specific binding effects at the sensor surface must also be carefully controlled, and this requires meticulous experimental design for SPR [12]. Electrochemical nucleic acid biosensors are based on the electrochemical behavior of the redox active nucleobases (e.g., guanine or adenine oxidation) or changes in the interfacial properties of the recognition layer or a signal change in the electroactive indicator agent after hybridization, which are the main approaches. Electrochemical biosensors do not suffer the drawback of high sensor setup complexity and cost. Other essential advantages of electrochemical biosensors are their robustness, easy miniaturization, sufficient detection limits, and requirement of low analyte volumes [13].

Graphene oxide (GO) has gained great attention in the field of biosensing [14]. Plenty of works based on incorporation of GO to the sensor surface in the absence or presence of other (nano)materials in a composite form have been reported so far [15,16,17,18]. For instance, Arfin and Rangari developed a ZnO-functionalized graphene oxide (GO)-modified glassy carbon electrode (GO–ZnO/GCE) used for the electrochemical sensory detection of phenol [15]. The cost-effective carbon monoxide sensor was developed on the interdigitated copper electrode based on PPy-GO composite materials by Farea et al. [16]. TiO_2_-reduced graphene oxide composite-modified glassy carbon electrodes (TiO_2_–ErGO–GCE) for the sensitive detection of tartrazine were reported by He et al. [17]. Ionic liquids (ILs) are considered as important alternative “Green” solvents with unique properties. Thus, ILs are attractive for a range of applications [19]. There are many biosensor studies that involve the use of various ILs as a supporting medium or an electrode modifier [20,21,22,23,24,25,26,27,28,29,30,31,32,33]. 

To date, numerous biosensors developed by using graphene-IL composites have been applied for detection of different analytes (i.e., small molecules). For example, Kim et al. [34] developed an electrochemical detection method for capsaicin using an IL-doped graphene-titania-nafion composite-modified electrode. Li et al. [35] designed a simple and sensitive electrochemical sensor based on an IL-functionalized graphene-oxide supported gold nanoparticles (GO-IL-AuNPs) coated glassy carbon electrode (GCE) for detection of dopamine. Ma et al. [36] developed a strategy for efficient determination of the sulfadiazine residues in the animal feed by applying the composite-modified electrode fabricated with the rGO and N-octyl-pyridinium-hexafluorophosphate (OPPF6) IL. On the other hand, Sukumaran et al. [37] developed a label-free platform based on an IL-functionalized nitrogen doped graphene (NrGO)-modified GCE for the discrimination of mutations in DNA. A hydrophilic IL was used to trap the DNA targets onto it by electrostatic interaction, and thereby it retained them close to the electrode surface suitable for the electroanalysis. The change in the peak current was explained based on the extent of the hindrance experienced by the redox species [Fe(CN)_6_]^3−^ for the electron transfer at various DNA modified electrodes. To the best of our knowledge, a GO-IL composite modified single-use graphite electrode was developed for the first time herein as a sensing platform and applied for detection of breast cancer 1 (BRCA1) gene through the direct measurement of the guanine oxidation signal. 

In this work, we aimed to develop a sensitive nucleic-acid biosensor related to the BRCA1 gene sequence. Under this goal, graphene-oxide and ionic liquid composite modified pencil graphite electrodes (GO-IL-PGEs) were developed for the first time herein as a single-use biosensing platform. The characterization of GO-IL modified electrodes was carried out with scanning electron microscopy (SEM), cyclic voltammetry (CV), and electrochemical impedance spectroscopy (EIS). Synthetic DNA sequences were used to elevate the experimental conditions as well as to test the selectivity of the biosensor. The hybridization detection was monitored by directly measuring the guanine oxidation signal via the differential pulse voltammetry (DPV) technique. 

## 2. Materials and Methods

### 2.1. Instrument

All measurements were performed by AUTOLAB-302 with NOVA 1.11 software (Eco Chemie, Utrecht, The Netherlands) in a Faraday cage with a three-electrode system (Figure S1). FRA module was used for impedimetric measurements. 

### 2.2. Chemicals

All oligonucleotides used in this study were acquired from TIB Molbiol (Berlin, Germany) (Table 1). Graphene oxide (GO) and 1-butyl-3-methylimidazolium hexafluorophosphate (IL) were acquired from DropSens (Spain) and Sigma-Aldrich, respectively. See the supplementary information for more information. 

Note that the BRCA1-specific DNA probe has an inosine (I) base instead of a guanine base since it is a synthetic analogue of a guanine base (Table 1). Based on this phenomenon, indicator-free detection of BRCA1 can be performed by directly measuring the guanine oxidation signal after full-match hybridization of the probe and the BRCA1 target [27,38].

**Figure 1:**
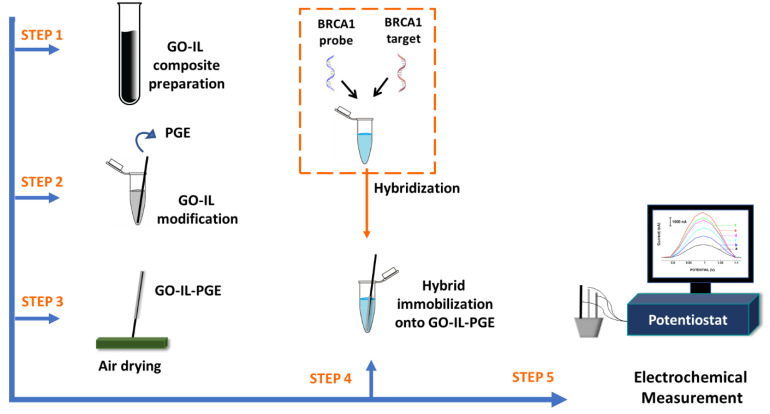
The schematic view of the experimental procedure followed for electrochemical sensing of BRCA1 gene using GO-IL-PGEs.

The detection protocol is given below and represented in Figure 1.

(i) GO-IL modification onto PGE surface.
(ii) Hybridization BRCA1 probe and BRCA1 target, or other DNA ODNs; NC, MM.
(iii) DNA–DNA hybrid immobilization onto GO-IL-PGE.

#### 2.2.1. Preparation of GO-IL-PGEs

GO was prepared in dimethyl sulfoxide (DMSO) as 50 µg/mL by following the sonication step during 30 min. The GO concentration was used in our study as 50 µg/mL, which was optimized in our previous work [39].

After sonication, IL was pipetted into the GO solution, and we obtained 7.5% IL in GO-IL solution and sonicated it for 30 min. PGEs were activated by applying +1.40 V for 30 s in ABS (pH 4.80) via DPV. These electrochemically activated PGEs were dipped into 100 µL of GO-IL and kept for 15 min to achieve the surface modification [39,40,41,42,43]. There was no precipitation in the solution of GO-IL observed during its immobilization onto the PGE surface. Then, GO-IL-PGEs were air-dried for 5 min.

#### 2.2.2. Hybridization of BRCA1-Specific DNA Probe with BRCA1 Target and Immobilization onto the GO-IL-PGEs

The BRCA1-specific DNA probe and the BRCA1 target were mixed and allowed for solution-phase hybridization during 5 min in PBS. Then, GO-IL-modified PGEs were dipped in vials containing BRCA1 probe-target hybrids and allowed for immobilization onto GO-IL-PGE surface during 30 min. Immobilization of hybrids arose by the formation of peptide bonds between 5′-end amino groups of BRCA1 probe-target hybrids and carboxyl groups of GO as reported in our previous study [39]. Due to this bond formation, no or negligible precipitation was expected during the sensing process. After 30 min of the immobilization step, the electrodes were washed with PBS to eliminate non-specific binding.

### 2.3. Voltammetric Measurements

The electrochemical characterization of electrodes was studied by using the CV technique. CVs were carried out by scanning between -0.8 V and +1.4 V with a 25 mV/s step potential and a 100 mV/s scan rate in the redox probe solution of 2.5 mM Fe(CN)_6_^3-/4-^ in 0.1 M KCl.

The guanine oxidation signal was measured by the DPV technique using the same conditions in our earlier studies [27,28,38]. DPVs were carried out by scanning with a 50 mV pulse amplitude and a 50 mV/s scan rate in ABS (pH, 4.80).

### 2.4. Impedimetric Measurements

The redox probe solution of 2.5 mM Fe(CN)_6_^3-/4-^ in 0.1 M KCl was used for EIS measurements. More information about impedimetric measurements is given in the supplementary information. 

## 3. Results and Discussion

**Figure 2:**
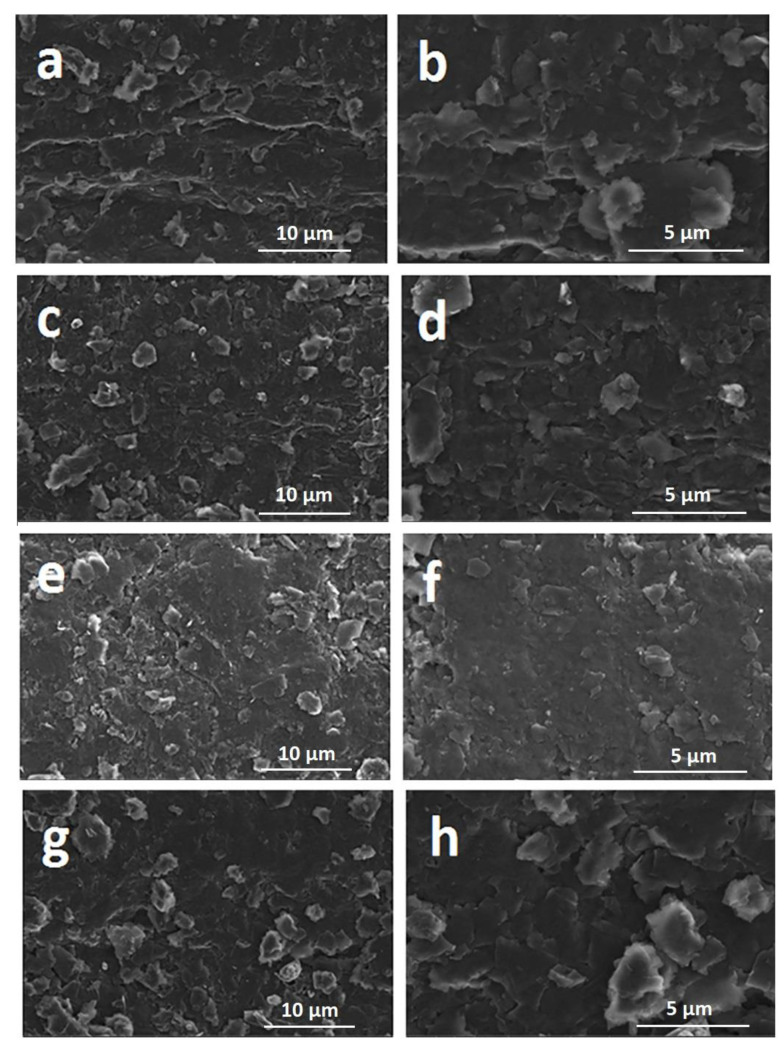
SEM images of (**a**,**b**) PGE, (**c**,**d**) GO-PGE, (**e**,**f**) IL-PGE, and (**g**,**h**) GO-IL-PGE with the resolutions of 10 and 5 µm.

The microscopic characterizations of PGE, GO-PGE, IL-PGE, and GO-IL-PGEs were recorded by a Quanta 400 FEI field emission scanning electron microscope (Tokyo, Japan) and are given in Figure 2. There are similarities between PGE (Figure 2a,b), GO-PGE (Figure 2c,d), and IL-PGE (Figure 2e,f), where the surface is relatively rough and layered. However, Figure 2g,h depicted SEM images of GO-IL-PGE, which was more laminated in contrast to the ones of GO-PGEs and IL-PGEs. 

**Figure 3:**
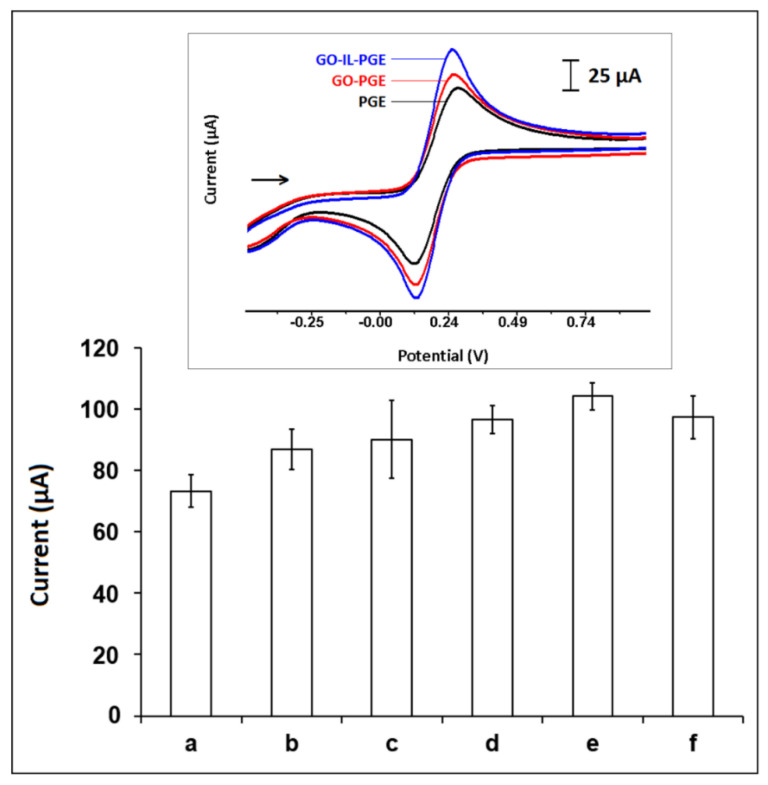
The average I_a_ values (*n* = 3) measured by (**a**) PGE and (**b**) GO-PGE and GO-IL-PGE in the presence of IL composition as (**c**) 2.5%, (**d**) 5%, (**e**) 7.5%, and (**f**) 10%. Inset: CVs recorded by PGE, GO-PGE, and GO-IL-PGE in the presence of an IL composition of 7.5%.

The effect of IL concentration was examined by measuring I_a_ values after modification of GO and IL at different percentages (2.5% to 10%) by the CV technique (Figure 3). Additionally, the electrochemical behavior of GO-IL-PGEs was investigated in comparison to PGE or GO-PGE. The corresponding anodic current (I_a_) and cathodic current (I_c_) with the relative charge Q (mC) and peak potential separation value (ΔE_p_) and the electroactive surface areas of PGE, GO-PGE, and GOL-IL-PGE are listed in Table 2. An increase was obtained at I_a_ when IL % was increased till 7.5% (Figure 3e), after then it decreased. As seen from Figure 3, the anodic and cathodic peak currents of GO-IL-PGE were higher than the ones measured by PGE or GO-PGE. This confirmed that the GO-IL expedited the electron convey between the electrode interface and the electrolyte. Additionally, the electroactive surface areas of the electrodes were calculated according to the Randles–Sevcik equation [44], and accordingly, the highest surface area (i.e., 0.314 cm^2^) was obtained in the case of GO-IL modification. The enhanced sensor response can be attributed to the acceleration of ion transportation from the electrolyte to the electrode surface in the presence of GO-IL in comparison to the one measured with PGE [27,28,29,39,40,41,42,43]. ΔE_p_ was found to be 151 mV by GO-IL-GE, while it was 173 mV with PGE. Thus, it was a quasi-reversible electrochemical response and convincing electrocatalytic effect of GO-IL modification toward the redox reaction of [Fe(CN)_6_]^3−/4−^. 

**Figure 4:**
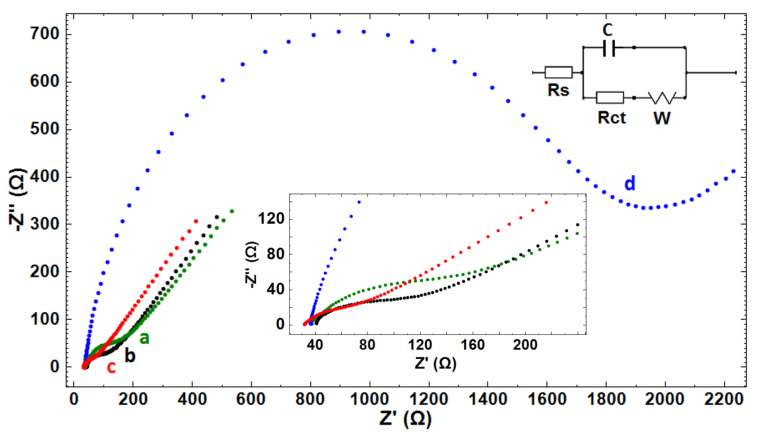
Nyquist diagrams of (**a**) PGE, (**b**) GO-PGE, (**c**) GO-IL-PGE, and (**d**) 100 µg/mL fsDNA immobilized GO-IL-PGE. Inset: The closer view of the Nyquist diagrams.

The electrochemical-impedance spectroscopy technique was used for stepwise characterization of GO-IL-PGEs before and after DNA immobilization. Figure 4 shows the impedimetric data obtained by PGE and GO-PGE and the results by using GO-IL-PGE before/after DNA immobilization. A standard Randles-equivalent circuit (Figure 4-inset) was used to fit EIS data. The Randles-equivalent circuit considers the resistance from the electrolyte solution (R_s_) and the double-layer capacitance (C) due to the accumulation of ions at the electrode surface as well as the charge-transfer resistance of the electrode surface (R_ct_) and the Warburg component (W) for diffusive proves [45]. Thus, the effect on the impedimetric response was elucidated by means of R_s_, R_ct_, C, and Warburg by fitting the experimental curves with the Randles-equivalent circuit. Further analysis of circuit components was extracted from the Randles-circuit model and is listed in Table 3. The average R_ct_ value of PGE was 73.30 ± 10.50 Ohm (RSD %, 14.30%, *n* = 3). In the presence of GO modification, a decrease in the R_ct_ value was observed since the GO-modified surface was becoming more conductive in contrast to the unmodified PGE, similarly as reported in the literature [38,39,40,43]. After GO-IL modification onto the electrode surface, there was a 43% decrease observed in the R_ct_ value as GO-IL composites improved conductivity at the electrode surface by facilitating more electron transfer between the solution and the electrode interface by graphene oxides, similarly as reported in earlier work [30]. 

There was an increase obtained in the R_ct_ value due to a repulsive interaction between the anionic redox probe and the negatively charged DNA-immobilized electrode [42,43]. Additionally, a decrease was observed in the C value after DNA immobilization, which is coherent with the theory that as a biomolecule binds it reduces the surface area by resulting with a 47% decrease in the capacitance. DNA immobilization onto the electrode surface also creates a shielding effect and lowers the columbic attraction between the electrode surface and the electrolyte [45]. Hence, a lower capacitance was recorded by DNA-immobilized GO-IL-PGE in contrast to bare GO-IL-PGE. By means of IL, an increase in the conductivity of the PGE surface can be obtained because of the increase in the electron transfer between the electrolyte and the electrode [29,41]. Regarding the results obtained by the electrode (PGE, GO-PGE, and GO-IL-PGE), there was a good agreement between the data recorded by the CV and the EIS techniques in our study.

**Figure 5:**
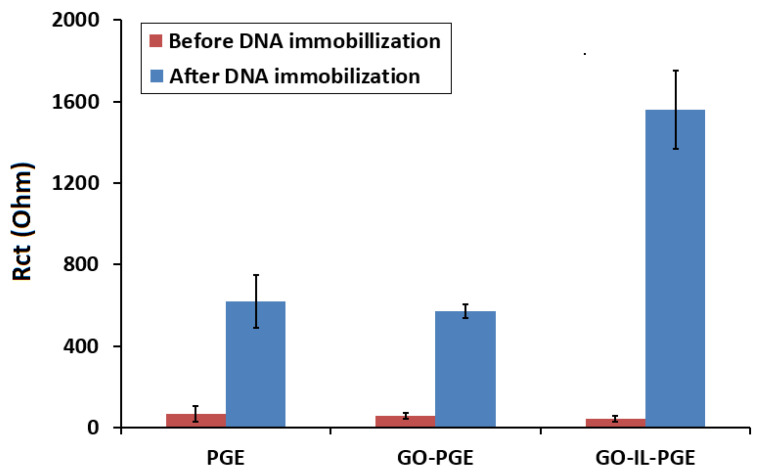
Histograms representing the average R_ct_ values before and after fsDNA immobilization onto the surface of PGE, GO-PGE, or GO-IL-PGE (*n* = 3).

In the presence of fsDNA immobilization on the PGE, the GO-PGE, or the GO-IL-PGE, the R_ct_ values were increased to 619 ± 130 Ohm (RSD %, 21%, *n* = 3), 570.50 ± 33.20 Ohm (RSD %, 5.80%, *n* = 3), and 1559.50 ± 193 Ohm (RSD %, 12.40%, *n* = 3), respectively (Figure 5). The increase in R_ct_ in case of fsDNA immobilization was due to the charge repulsion between the phosphate backbone of DNA and [Fe(CN)_6_]^3−/4−^, which resulted in a reduced ability for electron transfer at the electrode surface, as similarly reported in earlier works [42,43,46]. The highest increase in R_ct_ was obtained when fsDNA was immobilized onto the GO-IL-PGE relatively to the PGE or the GO-PGE. Furthermore, the apparent fractional coverage values ($\theta_{IS}^{R}$) for each of the electrode types were calculated by Janek et al.’s equation [47]. The $\theta_{IS}^{R}$ values for the fsDNA-immobilized PGE, GO-PGE, and GO-IL-PGE were found to be 0.881, 0.900, and 0.973, respectively. The highest $\theta_{IS}^{R}$ indicated successful sensor surface modification with GO-IL. 

**Figure 6:**
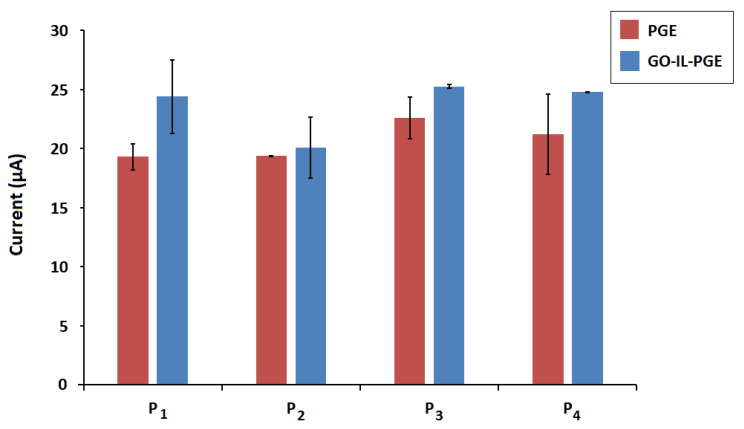
The average guanine oxidation signals (*n* = 3) obtained with P_1_-, P_2_-, P_3_-, and P_4_-immobilized PGE and GO-IL-PGE.

For better attachment of the DNA probe onto the sensor surface, the effect of 5′-end modification of the DNA probe was investigated. DNA probes with different 5′-end modifiers as amino, thiol, or phosphate were used and compared with the unmodified version of the probe (Figure 6). The guanine oxidation signals and RSD % values recorded by the PGE or the GO-IL-PGE are given in Table 4. The most reproducible and highest guanine signal with the relatively high increment % was obtained in the case of the amino-linked probe (P_4_) immobilization. Therefore, NH_2_ linkage was approved for effective probe immobilization onto the GO-IL-PGE surface.

**Figure 7:**
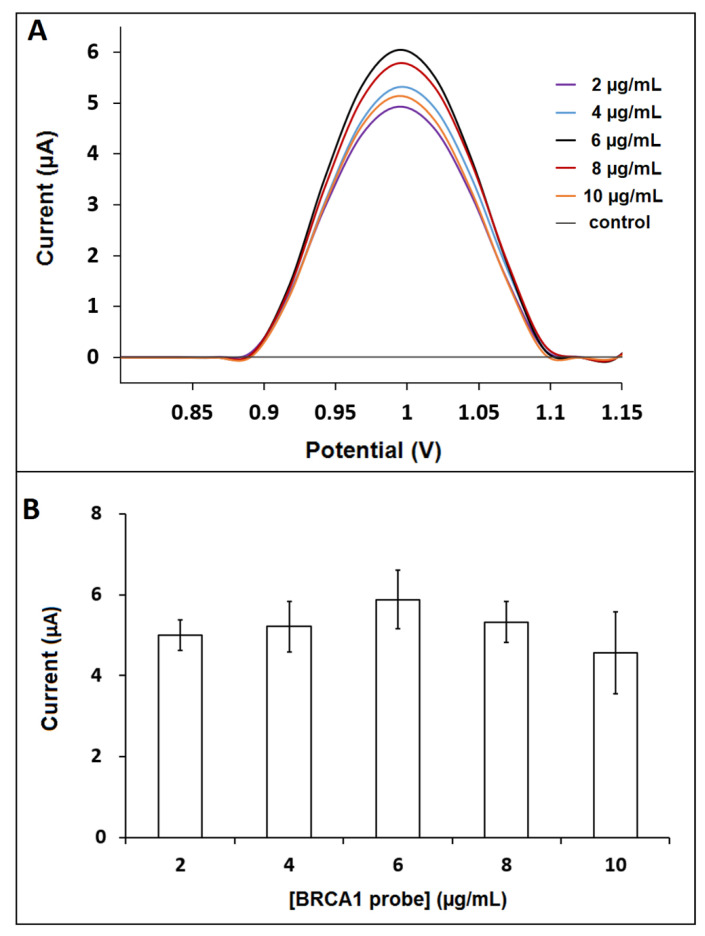
(**A**) DPVs recorded by GO-IL-PGE as control experiment in ABS; guanine oxidation signals after hybridization between the 2 µg/mL, 4 µg/mL, 6 µg/mL, 8 µg/mL, and 10 µg/mL BRCA1 probe and the 10 µg/mL BRCA1 target. (**B**) Histograms representing the guanine oxidation signals in case of hybridization between 2 to 10 µg/mL BRCA1 probe and BRCA1 target (*n* = 3).

The BRCA1 probe concentration was further optimized by testing five probe concentrations (from 2 to 10 µg/mL) (Figure 7). For this purpose, the solution-phase hybridization between the BRCA1 probe and the BRCA1 target was performed. After hybridization, the highest guanine oxidation signal was measured as 5.88 ± 0.73 µA (RSD %, 12.41%, *n* = 3), in the case of the 6 µg/mL BRCA1 probe (Figure 7).

**Figure 8:**
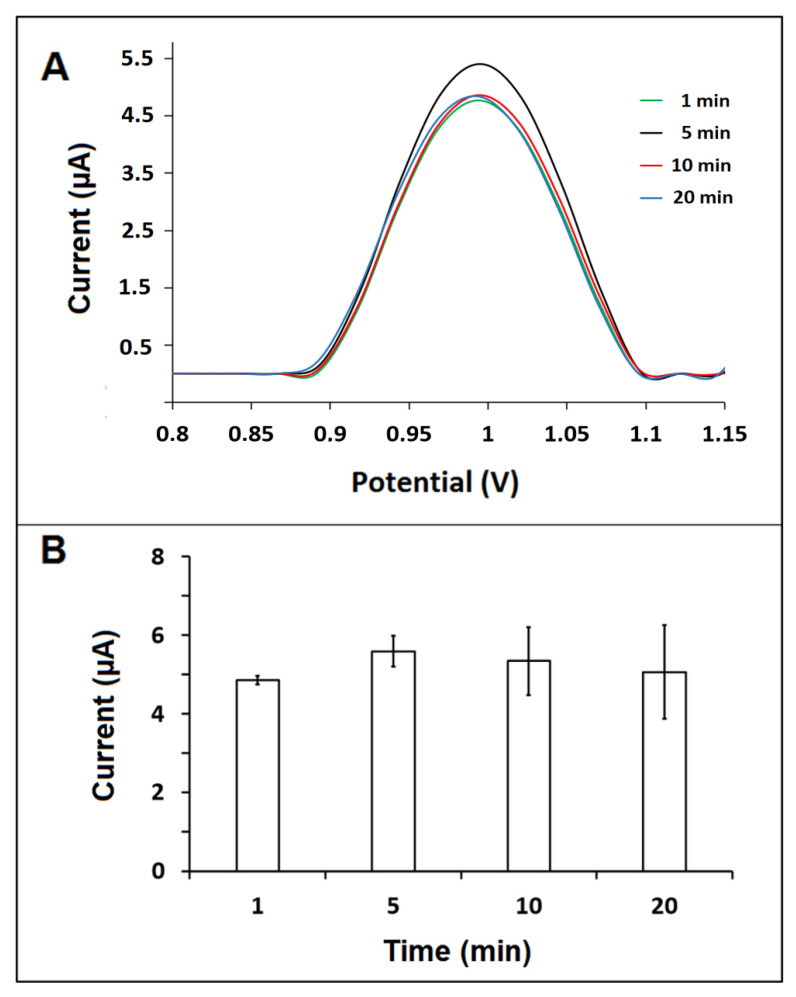
(**A**) DPVs. (**B**) The average guanine oxidation signals recorded by GO-IL-PGEs after hybridization of the 6 µg/mL BRCA1 probe with the 10 µg/mL BRCA1 target for 1, 5, 10, and 20 min (*n* = 3).

The hybridization between the probe and the BRCA1 target was performed during 1, 5, 10, and 20 min. The guanine signal was recorded as 5.58 ± 0.39 µA (RSD %, 7.05%, *n* = 3), which was the highest signal when hybridization was performed during 5 min (Figure 8). Therefore, the optimum hybridization time was chosen as 5 min.

**Figure 9:**
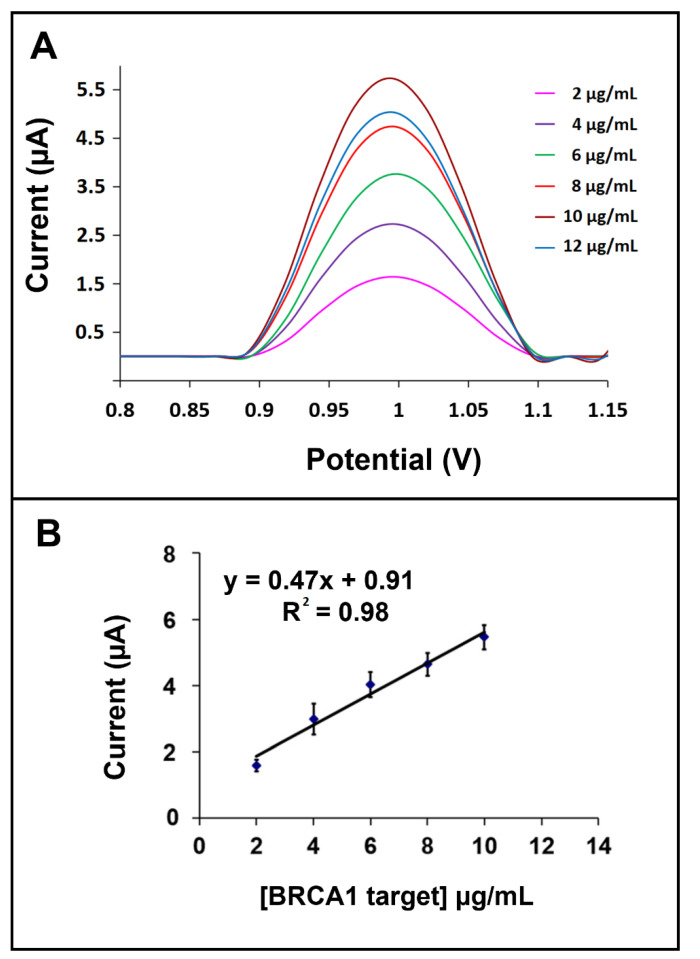
(**A**) DPVs. (**B**) Calibration plot with the average guanine signal of hybridization between the 6 µg/mL BRCA1 probe and the 2 µg/mL, 4 µg/mL, 6 µg/mL, 8 µg/mL, and 10 µg/mL BRCA 1 target on the surface of GO-IL-PGE (*n* = 3).

The effect of the BRCA1 target concentration at the hybridization process was then investigated. Firstly, the hybridization between the 6 µg/mL BRCA1 probe and different concentrations of the BRCA1 target from 2 to 12 µg/mL was performed. The signal was increased up to 10 µg/mL; then, it was fixed (shown in Figure S2). Therefore, the 10 µg/mL BRCA1 target concentration was chosen as the optimum. The limit of detection (LOD) was calculated in the concentration range of 2–10 μg/mL with the equation y = 0.47x + 0.91 and R^2^ = 0.98 (shown in Figure 9B), and the LOD was found to be 1.48 µg/mL [48]. Additionally, the sensor sensitivity was estimated from the slope of the calibration curve divided by the surface area of the GO-IL-PGE and was found to be 1.49 µA.mL/µg.cm^2^.

**Figure 10:**
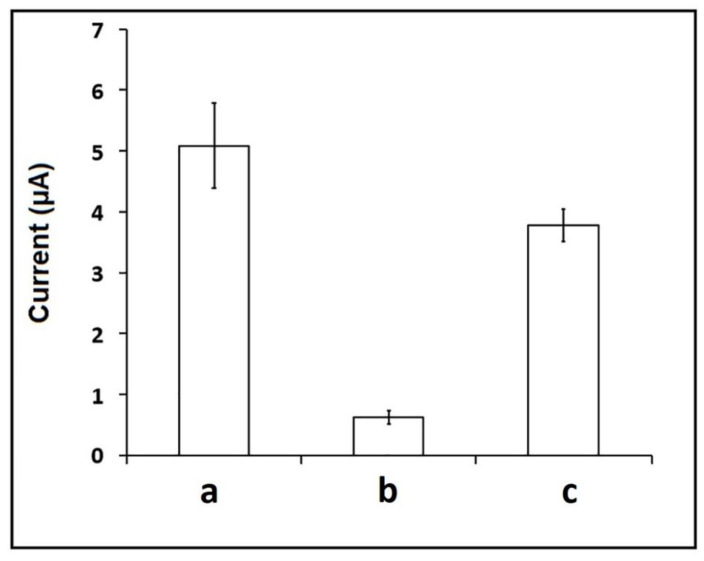
The average guanine signal (*n* = 3) after hybridization between BRCA1 probe and BRCA1 target (**a**), or MM (**b**), or mixture of BRCA 1 target and MM (**c**).

The selectivity of the assay was tested in case of hybridization between the probe and the BRCA1 target or 3-base mismatched (MM) or the mixture samples containing target:MM. The highest guanine signal was obtained with the full-match hybridization between the BRCA1 probe and the target (Figure 10) and was measured as 5.08 ± 0.69 µA (RSD %, 13.74%, *n* = 3). The negligible guanine oxidation signal was 0.62 ± 0.10 µA (RSD %, 17.32%, *n* = 3) in case of hybridization between the BRCA1 probe and the MM sequence. Otherwise, in the case of hybridization between the probe and the BRCA1 target in the presence of MM sequence, almost the same guanine signal was measured with the one obtained by the full-match hybridization signal. Therefore, it can be concluded that our assay presented a selective behavior on BRCA1-detection.

The intra-day repeatability of the assay by GO-IL-PGEs was examined by measuring the guanine oxidation signal in the case of hybridization between the probe and the 10 µg/mL BRCA target consecutively during three days (Table S1). The values of RSD % varied from 0.8% and 8.8%. In order to examine the inter-day reproducibility, these results were combined, and the average signal was calculated and found to be 5.01 ± 0.33 µA with a RSD % of 6.62% (*n* = 7) (shown in Table S2). The results revealed that the GO-IL-PGEs exhibited a satisfactory reproducibility with a mean change in the response of 0.33 µA and a relative standard deviation of 6.62% (*n* = 7).

## 4. Conclusions

In this study, graphene-oxide (GO) and ionic-liquid (IL) composite-modified pencil-graphite electrodes (GO-IL-PGEs) were developed for the first time and then applied as a biosensing platform for electrochemical detection of breast cancer 1 (BRCA1) gene. The detection protocol consists of GO-IL modification onto the PGE surface, hybridization of a BRCA1-specific probe with its target, and finally the immobilization of DNA–DNA hybrid onto the surface of the GO-IL-PGE. The GO-IL incorporated the advantages of GO and IL, which provide a thin film at the electrode surface for immobilization of the BRCA1 probe–target hybrid. Additionally, the GO-IL composite could offer enhanced electrocatalytic properties and a larger electroactive surface area than the bare electrode or only the GO-modified electrode. The hybridization was monitored by directly measuring the guanine oxidation signal at +1.0 V using the differential pulse voltammetry (DPV) technique. In our previous work [41], chemically activated and graphene oxide (GO) modified graphite electrodes (CA-GO-PGEs) were used for impedimetric detection of miRNA-34a, which was a biomarker of Alzheimer’s disease. In that assay, the PGE surface was firstly activated by using covalent agents (CA), and then GO modification was done by itself. In the meantime, hybridization of the miRNA-34a-specific DNA probe with the miRNA-34a RNA target was performed in the solution phase. After all, the sample containing DNA–miRNA hybrids were immobilized onto the CA-GO-PGE surface. The impedimetric detection of miRNA-34a was then performed by measuring the fractional change at the charge transfer resistance (R_ct_). The aim of the earlier work was to develop an impedimetric assay for the detection of microRNA by GO-modified electrodes. On the other hand, the aim of our present work was to develop a voltammetric assay in combination with GO-IL-composite-modified electrodes to detect breast cancer 1 (BRCA1) gene while incorporating the advantages of these two materials while increasing the electrocatalytic properties of the electrode. The proposed assay was able to identify the complementary target sequence even in the mixture with mismatched sequences. The preparation of GO-IL-PGEs was performed in 20 min, and BRCA1 gene detection could be managed within 35 min with a LOD of 1.48 μg/mL (equal to 251 nM or 25 pmol in a 100 μL sample). Some of the earlier studies related to the electrochemical detection of BRCA1 gene were summarized in Table 5 [49,50,51,52,53,54,55,56,57]. 

Although lower LOD values have been reported in previous studies [50,51,52,53,54,56,57], the LOD value obtained herein was in the acceptable level and was even lower than the ones reported by some of the earlier studies [49,55]. The single-use GO-IL-modified sensors brought herein the following important advantages: they are easy to use and cost effective per measurement, resulting with reproducible results in a short time (35 min) in comparison to earlier works. Therefore, in comparison to the conventional nucleic acid detection technologies, our assay provides a rapid, practical, low-cost, and user-friendly nucleic acid analysis with a satisfactory detection limit [2,3,4,5,6]. 

With any of the conventional methods (e.g., capillarity electrophoresis, sequencing, and PCR-mediated techniques) BRCA1 gene detection could be achieved in a short time; however, the determination was carried out in 35 min in our electrochemical DNA biosensor-based assay. This simple modification and detection protocol can be extended for the monitoring of different type of nucleic acids or protein detection further, which can prompt new opportunities to design novel nucleic-acid-sensing strategies and applications.

## Figures and Tables

**Table 1:** The base sequences of all oligonucleotides.

Oligonucleotide	Sequence
Probe (P_1_)	5′-(CH_2_)_6_-GGGGGGGGGGGG 3′
SH-link probe (P_2_)	5′-SH-(CH_2_)_6_-GGGGGGGGGGGG-3′
PO_4_ link probe (P_3_)	5′-PO_4_-(CH_2_)_6_-GGGGGGGGGGGG-3′
NH_2_ link probe (P_4_)	5′-NH_2_-(CH_2_)_6_-GGGGGGGGGGGG-3′
BRCA1 probe (I: Inosine)	5′-NH_2_-(CH_2_)_6_-IATTTTCTTCCTTTTITTC-3′
BRCA target	5′-GAACAAAAGGAAGAAAATC-3′
3-base mismatch (MM)	5′-CAACAAAAGCAACAAAATC-3′
Non-complementary (NC)	5′-TAAGCAACCTGATTTGA-3′

**Table 2:** The average values (*n* = 3) of anodic and cathodic peak currents (I_a,_ I_c_), relative anodic and catodic charge (Q_a,_ Q_c_), and ΔE_p_ values and electroactive surface areas (A) for PGE, GO-PGE, and GO-IL-PGE (in case of 7.5% IL concentration).

Electrodes	I_a_ (µA)	I_c_ (µA)	Q_a_ (mC)	Q_c_ (mC)	ΔE_p_ (mV)	A (cm^2^)
PGE	73.22 ± 5.34	77.93 ± 6.88	1.73	0.97	0.173	0.221
GO-PGE	86.78 ± 6.61	90.63 ± 10.09	2.18	1.11	0.139	0.261
GO-IL-PGE	104.09 ± 4.37	100.61 ± 3.82	2.18	1.14	0.151	0.314

**Table 3:** EIS response parameters obtained after curve fit of the Nyquist diagrams presented in Figure 4 using Randles circuit.

Electrode	R_s_ (Ohm)	R_ct_ (Ohm)	C (µF)	W (mMho)
PGE	31.9	70.9	12.1	2.39
GO-PGE	40.8	48.3	6.57	2.14
GO-IL-PGE	29.5	43.2	11.7	2.72
DNA-immobilized GO-IL-PGE	37.8	1420	6.23	1.18

**Table 4:** The average guanine oxidation signals (*n* = 3) and RSD % values recorded by PGE or GO-IL-PGE in case of immobilization of probe (P_1_), -SH linked (P_2_), -PO_4_ linked (P_3_), and -NH_2_ linked probe (P_4_). Increase % values were calculated by computing the difference between the signals recorded by PGE and GO-IL-PGE and comparing that difference to the signal recorded by PGE.

Probe	Electrode	Current (µA)	RSD (%)	Increase (%)
**P_1_**	PGE	19.30 ± 1.12	5.85	26.4
	GO-IL-PGE	24.40 ± 3.10	12.73	
**P_2_**	PGE	19.36 ± 0.05	0.26	3.74
	GO-IL-PGE	20.09 ± 2.60	12.97	
**P_3_**	PGE	22.60 ± 1.76	7.82	11.77
	GO-IL-PGE	25.26 ± 0.16	0.65	
**P_4_**	PGE	21.20 ± 3.40	16.05	17.05
	GO-IL-PGE	24.80 ± 0.01	0.03	

**Table 5:** The comparison of studies developed for electrochemical detection of BRCA1 gene.

Electrode	Modification	Method	LOD	Reference
SPE	SWCNT	EIS and DPV	378.52 nM	[49]
GCE	GO/AuNP	CV and chronoamperometry	1 fM	[50]
AuE	MCH	EIS and DPV	0.05 nM	[51]
-	GO		0.2 nM	[52]
GCE	MWCNT	CV and chronoamperometry	2 nM	[53]
AuE	Zwitterionic peptide	EIS	0.3 fM	[54]
AuE	ZnONWs	DPV	3.32 μM	[55]
GCE	(SIL-g-(N)GAs)	CV and DPV	3 pM	[56]
SPE	AuNPs	CV and amperometry	0.1 fM	[57]
PGE	GO-IL composite	DPV	251 nM	This work

**Abbreviations:** SPE: screen-printed electrode, GCE: glassy carbon electrode, AuE: gold electrode, SWCNT: single-wall carbon nanotube, AuNP: gold nanoparticles, MCH: mercaptohexanol, MWCNT: multi-wall carbon nanotube, ZnONWs: zinc-oxide nanowires, (SIL-g-(N)GAs): supramolecular ionic liquids grafted on nitrogen-doped graphene aerogels.

## Data Availability

The data presented in this study are available within the article and its Supplementary Materials. Other data that support the findings of this study are available upon request from the corresponding author.

## Supplementary Materials

The following supporting information can be downloaded at: https://www.mdpi.com/article/10.3390/bios12020095/s1, Figure S1: The electrochemical cell with three-electrode setup including PGE as a working electrode, silver chloride electrode as reference and platinum wire as counter electrode during measurement. Left image represents the enlarged view of the electrochemical cell with three-electrode system, Figure S2: Line graph with the average guanine signal of hybridization between 6 µg/mL BRCA1 probe and 2 µg/mL, 4 µg/mL, 6 µg/mL, 8 µg/mL, 10 µg/mL, 12 µg/mL BRCA 1 target on the surface of GO-IL-PGE (*n* = 3), Table S1: The guanine oxidation signals measured in the presence of the hybridization between probe and 10 μg/mL BRCA1 target by GO-IL-PGEs for three different days with the values of the average guanine oxidation signals (*n* = 2 or *n* = 3) and the standard deviation with the RSD % for presenting the intra-day reproducibility, Table S2: The guanine oxidation signals measured in the presence of the hybridization between probe and 10 μg/mL BRCA1 target by GO-IL-PGEs for three different days with the average guanine oxidation signals (*n* = 7) and the standard deviation with the RSD % for presenting the inter-day reproducibility.
