# Supplementary material for: Graphene-Oxide and Ionic Liquid Modified Electrodes for Electrochemical Sensing of Breast Cancer 1 Gene
Source: Biosensors (Basel). 2022 Feb 4;12(2):95. doi: 10.3390/bios12020095 (PMC8870019; doi:10.3390/bios12020095)
Supplement: Supplementary file 1 [file biosensors-12-00095-s001.zip › biosensors-1551736-SI.pdf]

Supplementary Material

Graphene oxide / ionic liquid modified electrodes for electrochemical sensing of breast cancer 1 gene

Preparation of oligonucleotides

All stock solutions of oligonucleotides (500 µg/mL) were prepared with Tris–EDTA buffer (10 mM Tris–HCl, 1 mM EDTA, pH 8.00) and kept at -20 ºC. Diluted solutions of oligonucleotides were prepared with 50 mM phosphate buffer containing 20 mM NaCl (PBS, pH 7.40). The fish sperm DNA (fsDNA) was dissolved in ultrapure water and kept frozen. Diluted solutions of fsDNA were prepared in 0.50 M acetate buffer solution containing 20 mM NaCl (ABS, pH 4.80).

All other chemicals were of analytical reagent grade and were supplied from Sigma and Merck. All other stock solutions were prepared using ultrapure and deionized water.

Impedimetric measurements

The impedance was measured in the frequency range from 100 mHz to 1 kHz at open circuit potential of +0.23 V versus Ag/AgCl/3 M KCl with a sinusoidal signal of 10 mV. The real and imaginary impedance (Z' and -Z'') are the components of the complex impedance (Z). An equivalent circuit model (Randles circuit) was utilized for fitting of impedimetric results. The respective semicircle diameter corresponds to the charge-transfer resistance, R_{ct}, the values of which are calculated using the fitting program AUTOLAB 302 NOVA 1.11 (FRA, version 2.0 Eco Chemie, The Netherlands).

A Randles circuit used to fit impedance data was given as inset of all Nyquist diagrams.

The electron transfer was limited at higher frequencies and the linear section seen at lower frequencies may be attributed to the diffusion as explained earlier studies [1]. $\boldsymbol{R_S}$ is the solution resistance. The constant phase element $\boldsymbol{Q}$ is then related to the double layer capacitance at the electrode-electrolyte interface. $\boldsymbol{R_{ct}}$ is related to the charge transfer resistance at the electrode-electrolyte interface. The constant phase element $\boldsymbol{W}$ is the Warburg impedance due to mass transfer to the electrode surface.

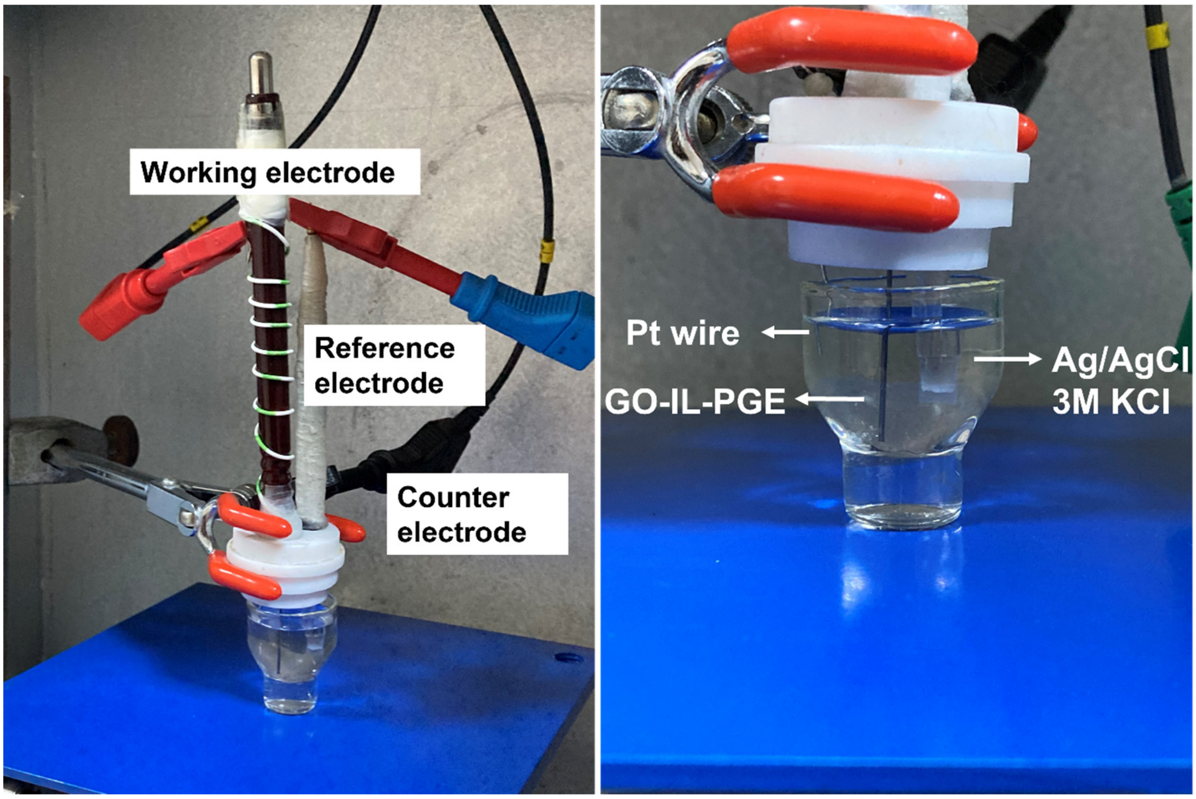

Figure S1. The electrochemical cell with three-electrode setup including PGE as a working electrode, silver chloride electrode as reference and platinum wire as counter electrode during measurement. Left image represents the enlarged view of the electrochemical cell with three-electrode system.

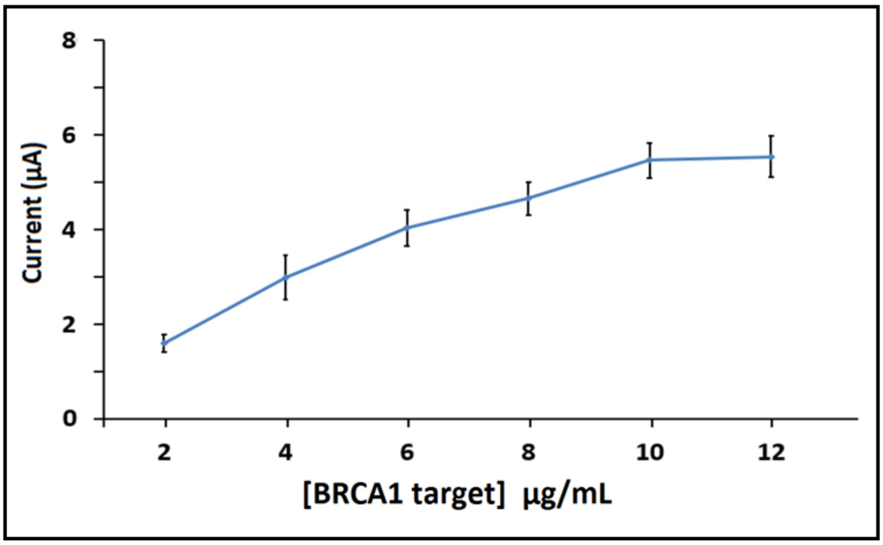

Figure S2. Line graph with the average guanine signal of hybridization between 6 μg/mL BRCA1 probe and 2 μg/mL, 4 μg/mL, 6 μg/mL, 8 μg/mL, 10 μg/mL, 12 μg/mL BRCA 1 target on the surface of GO-IL-PGE (n=3).

Table S1. The guanine oxidation signals measured in the presence of the hybridization between probe and 10 µg/mL BRCA1 target by GO-IL-PGEs for three different days with the values of the average guanine oxidation signals (n=2 or n=3) and the standard deviation with the RSD % for presenting the intra-day reproducibility.

	**Intra-day reproducibility**		
	**1st day**	**2nd day**	**3rd day**
	5.14	4.60	4.92
	5.08	5.32	5.40
		4.59	
**Average (µA)**	**5.11**	**4.83**	**5.16**
**Standard Deviation**	**0.04**	**0.42**	**0.34**
**RSD %**	**0.87**	**8.88**	**6.67**

Table S2. The guanine oxidation signals measured in the presence of the hybridization between probe and 10 µg/mL BRCA1 target by GO-IL-PGEs for three different days with the average guanine oxidation signals (n=7) and the standard deviation with the RSD % for presenting the inter-day reproducibility.

	**Inter-day reproducibility**	
	5.14	1st day
	5.08	
	4.60	2nd day
	5.32	
	4.59	
	4.92	3rd day
	5.40	
**Average (µA)**	**5.01**	
**Standard Deviation**	**0.33**	
**RSD %**	**6.62**	

References

[1] F. Lisdat, D. Schafer, The use of electrochemical impedance spectroscopy for biosensing, Anal. Bioanal. Chem. 391 (2008) 1555-1567.
